# The exploration of remote simulation strategies for the acquisition of psychomotor skills in medicine: a pilot randomized controlled trial

**DOI:** 10.1007/s44217-023-00041-2

**Published:** 2023-07-17

**Authors:** Natasha Guérard-Poirier, Léamarie Meloche-Dumas, Michèle Beniey, Andrei Torres, Bill Kapralos, Malek Dhane, Frédéric Mercier, Rami Younan, Adam Dubrowski, Erica Patocskai

**Affiliations:** 1 Faculty of Medicine, Université de Montréal, Montreal, QC H2X3E4 Canada; 2 Department of Surgery, Université de Montréal, Montreal, QC H2X3E4 Canada; 3 Faculty of Business and IT, OntarioTech University, Oshawa, ON L1G 0C5 Canada; 4 Faculty of Health Sciences, OntarioTech University, Oshawa, ON L1G 0C5 Canada; 5 Department of Surgical Oncology, Centre Hospitalier de L’Université de Montréal, 1051 Rue Sanguinet, Montreal, QC H2X3E4 Canada

**Keywords:** Decentralized simulation, Peer learning, Simulation in surgery, Subcuticular sutures, Surgical education

## Abstract

**Background:**

Progress in remote educational strategies was fueled by the advent of the COVID-19 pandemic. This pilot RCT explored the efficacy of a decentralized model of simulation based on principles of observational and peer-to-peer learning for the acquisition of surgical skills.

**Methods:**

Sixty medical students from the University of Montreal learned the running subcuticular suture in four different conditions: (1) Control group (2) Self-learning (3) Peer-learning (4) Peer-learning with expert feedback. The control group learned with error-free videos, while the others, through videos illustrating strategic sub-optimal performances to be identified and discussed by students. Performance on a simulator at the end of the learning period, was assessed by an expert using a global rating scale (GRS) and checklist (CL).

**Results:**

Students engaging in peer-to-peer learning strategies outperformed students who learned alone. The presence of an expert, and passive vs active observational learning strategies did not impact performance.

**Conclusion:**

This study supports the efficacy of a remote learning strategy and demonstrates how collaborative discourse optimizes the students’ acquisition of surgical skills. These remote simulation strategies create the potential for implantation in future medical curriculum design.

*Trial Registration*: NCT04425499 2020-05-06.

## Introduction

Surgical skills simulation laboratories support the development of technical and non-technical surgical skills for professional practice [1]. They are experiential classrooms embedded within medical schools and, or hospitals where learners (e.g., medical students and surgical residents) acquire a range of skills in an environment that offers the practicality of a surgical setting without the risks to patient safety. In this study, we focus on a learning environment for technical skills referred to as the centralized model of simulation (C-SIM). C-SIM is characterized by an environment for learners to practice technical surgical skills under the supervision of experienced educators [1]. The typical training session in the Ce-SIM model consists of three main phases: instructions and preparation, hands-on practice, and practice/post-practice feedback.

Although the idea of a decentralized model of simulation (DeC-SIM) has been investigated for a few decades [2], the recent COVID-19 pandemic has catalyzed these efforts [3, 4]. During the COVID-19 pandemic, the access to surgical skills simulation laboratories became limited due to physical distancing, and in order to continue skills development, other options needed to be considered [4]. As opposed to C-SIM, DeC-SIM is characterized by an environment in which learners can prepare, practice, and receive feedback remotely and outside of the simulation laboratories from the comfort of their homes or other locations.

The overarching theory used to guide the development of technical surgical skills is Ericsson’s deliberate practice [5]. It refers to a particular type of practice that is purposeful and systematic through the use of instructions, motivation, and accurate feedback [1, 4, 5]. There are several instructional design elements that need to be addressed through research, before educators and program directors were to consider DeC-SIM as a possible augmentation to more traditional training approaches in the post-pandemic era. These instructional design elements need to apply to all three phases of simulation (i.e., instructions and preparation, hands-on practice, and practice/post-practice feedback). In this study, we address how to structure *instructions* and *pre-practice preparation* in a DeC-SIM model to (a) most optimally develop procedural knowledge prior to physical practice, and (b) improve learners’ performance in the initial hands-on practice. Evidence suggests that trainees can acquire suturing skills independently [6–9], however, the efficacy of this type of practice is influenced by how well these trainees were instructed [10, 11]. That is, the efficacy of hands-on practice depends on the efficacy of instructions and preparation [11]. Furthermore, it has been shown that surgical trainees are effective at using video-based instructions for preparation [12], and that creating opportunities for peer-to-peer collaboration [13, 14], with and without an expert [2, 15], can further facilitate preparation and effective instructions. However, these isolated instructional elements have not been put together into a more complex educational intervention that would support the DeC-SIM model in the future [16–18]. Therefore, the purpose of this study was to investigate the efficacy of a complex educational intervention as a means to prepare senior medical students for subsequent hands-on, simulation-based practice in a DeC-SIM model of simulation.

## Materials and methods

This study was approved by the institutional review board of the University of Montreal (CERSES 20-068-D); registered (ClinicalTrials.gov NCT04425499); and completed in 2020 as a pilot randomized control, four-arm experimental design. This study was conducted in accordance with the principles of the international conference on the harmonization of the guidelines of good clinical practice (International Conference on Harmonization Guidelines for Good Clinical Practice (ICH-GCP)) and following the declaration of Helsinki on human research.

### Consent statement

Informed consent (consent to participate and consent for the results to be published) were obtained from all participant. All participants were over 18 years old.

### Participants

Sixty (n = 60) first—(n = 43) and second—(n = 17) year medical students were recruited to voluntarily participate in this study. The only inclusion criterion was an active enrollment within the first 2 years of medical school. A short questionnaire inquiring about previous experience was completed by the participants in order to confirm their level was novice. Exclusion criteria were: Self-reported injury during the trial; completing surgical rotations before the trial; returning from a break such as a sabbatical, and a medical degree in another country. The participants could withdraw from the study at any point and have their data excluded. After informed consent, all participants were randomly assigned to four experimental groups using stratified randomization (by year of study). This occurred via a pseudo-random number generator in which a default seed was used as the reference for randomness, assigning the participant to one of the four groups. When a group reached the target number of participants (n = 15) the assignment was stopped.

### Power calculation

Using global rating scale (GRS) scores [19] from previous work [6, 20], and based on 0.8 power to detect a statistically significant difference (p = 0.05, two-sided), 12 students per group were the minimum required.

### Materials and instruments

**Fig. 1:**
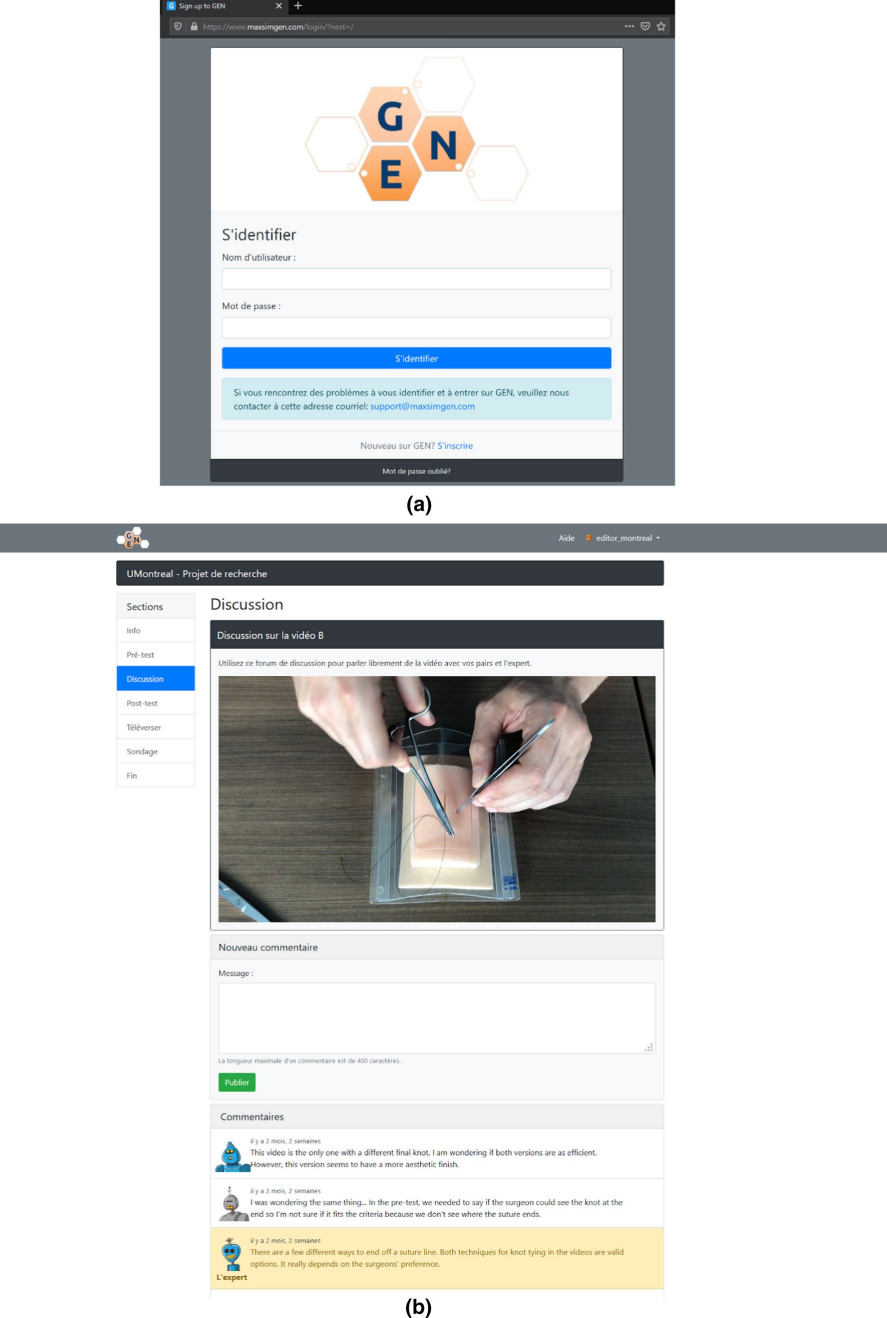
This figure illustrates a screen capture for the **a** log in page and **b** working space for the GEN learning management system used by the participants to learn the skills

*The Gamified Educational Network (GEN) Learning Management System (LMS)* is a multi-feature, online learning management system developed at maxSIMhealth laboratory (maxSIMhealth.com), Ontario Tech University that combines online learning and home-based simulation [4]. GEN permits easy content creation and integration of features such as instructions, collaboration, video uploads, and feedback through video assessment. Several features utilized in this study include (Fig. 1): upload feature, where the participants upload a video of themselves performing the suturing skill; a collaborative discussion board, which permits collaboration and feedback, and multiple choice surveys in the form of the global rating scales (GRS and checklists) adjacent to the videos. GEN displays segmented progression bars and permits selective section completion, where progressive completion blocks sections and guides the participants through the activity. For this study, GEN was made available in French and English and was designed to be platform agnostic (i.e., accessible by desktop computers, tablets, and smartphones).

#### Simulators and instruments

**Fig. 2:**
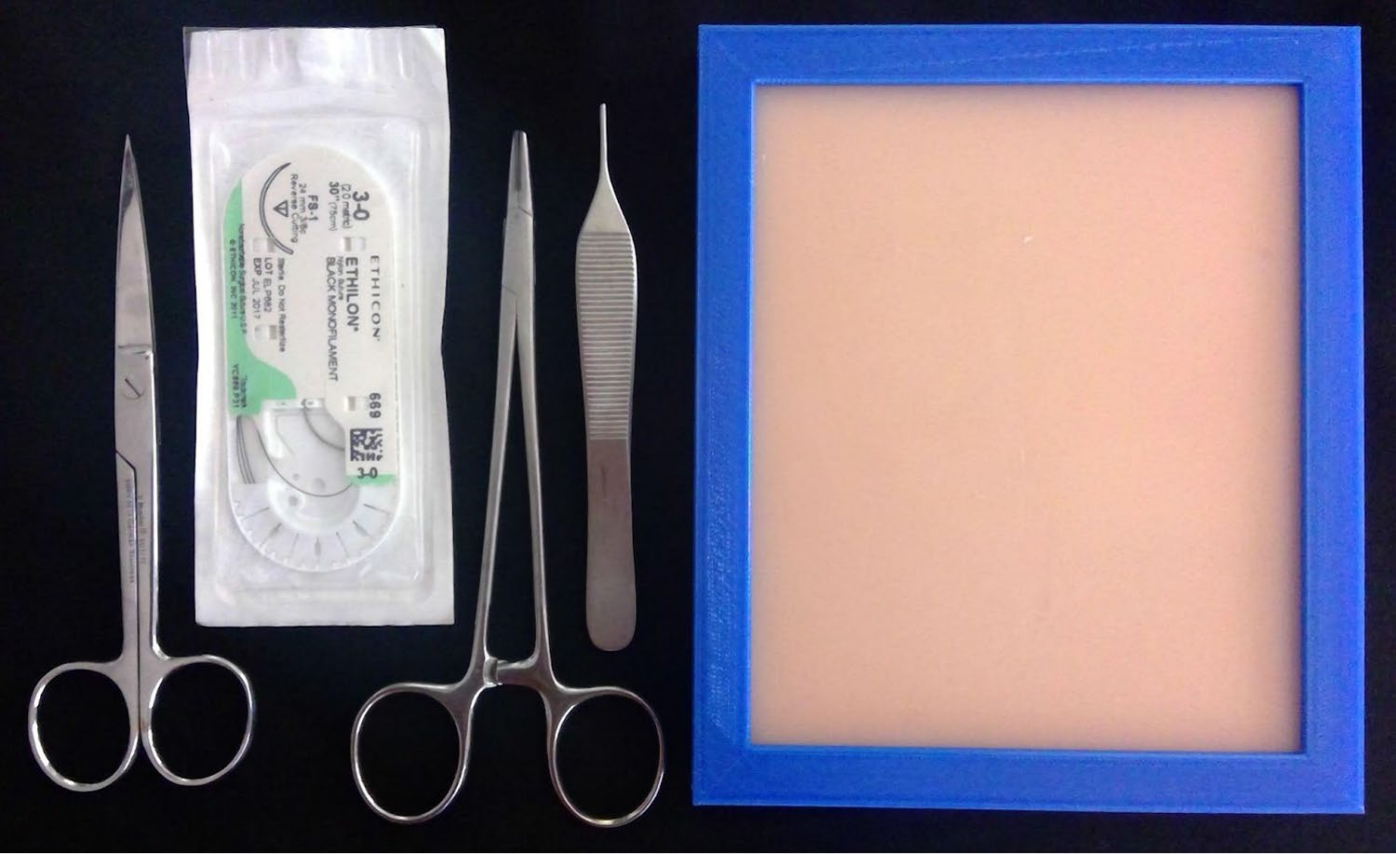
This figure illustrates the material that comprised the suturing kit the students received by mail, as a home-based simulator

The simulators were suturing pads (FAUX Skin Pad, https://www.fauxmedical.com/) affixed to a table's surface using a custom designed holder (maxSIMhealth.com). The sutures (3–0) and instruments (needle driver, forceps, scissors) were supplied by the *Unité de formation chirurgicale* of the *Hôpital Maisonneuve-Rosemont*, Montreal (Fig. 2).

### Procedure

**Fig. 3:**
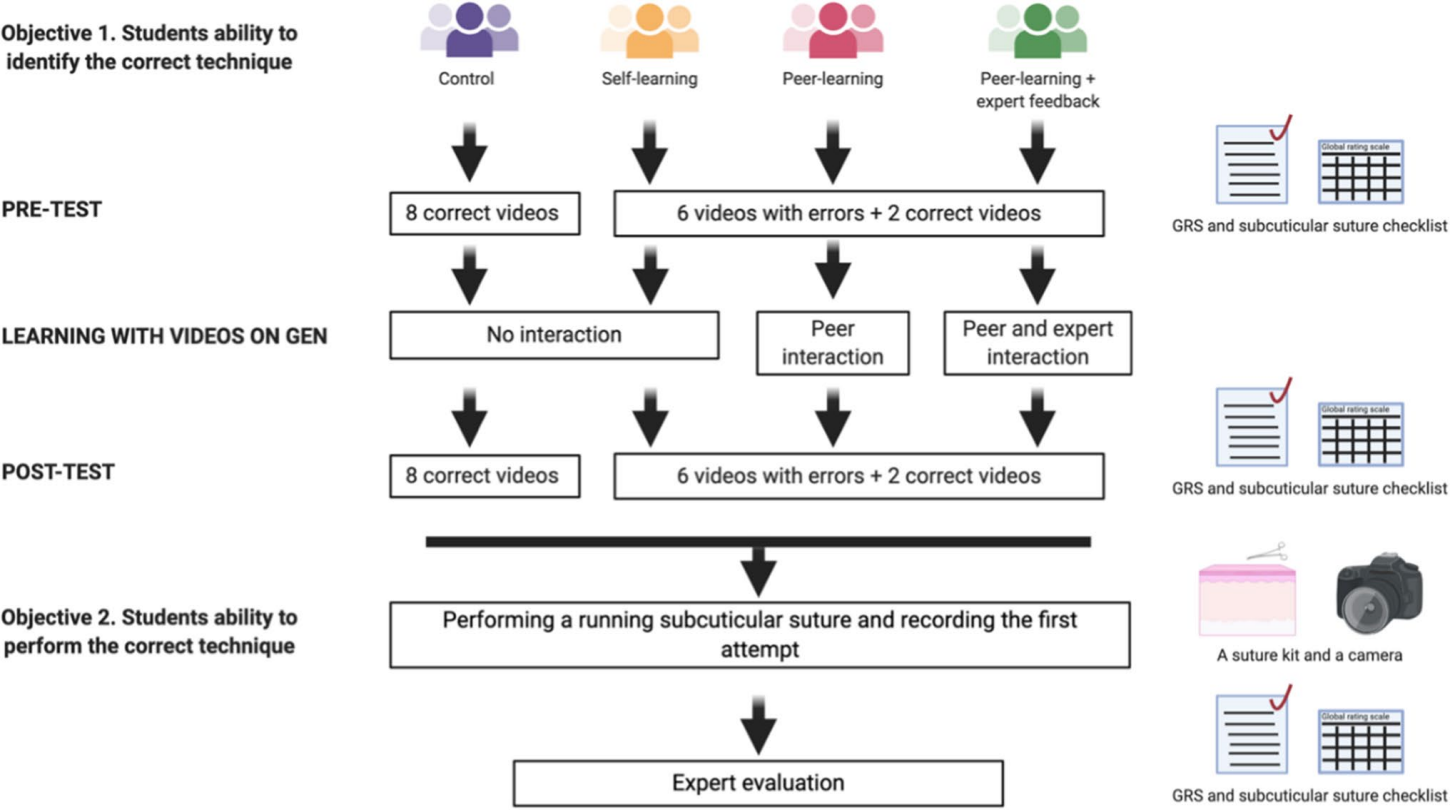
This figure demonstrates the study design

The protocol of this study has been previously published [21], (Fig. 3). The study was divided into two distinct phases. The first phase was the acquisition of procedural knowledge. The main aims of this phase were to assess the learners’ procedural knowledge (pre-test), provide them with opportunities for observational practice, collaboration and feedback, and re-test their procedural knowledge (post-test). The observational practice, collaboration and feedback sub-phase were constructed based on the Cheung et al. study [2]. The second phase was a test of initial hands-on performance. This phase was based on Miller’s model and assessed the ‘procedural knowledge’ (knows how), and ‘competence’ (shows how) [22]. These two levels were included as the overarching goal was to test how well, in the absence of physical practice, the elements of observational practice, collaboration, and feedback, prepare learners for subsequent practice in simulation.

#### Phase 1: The acquisition of procedural knowledge

This first part of the study includes the pre- and the post-test which focuses on knowledge acquisition. On the first day of the study, participants were emailed a unique link to access GEN. They were directed to the introduction page which briefly explained the activities, their due dates, and the steps required to complete the project (refer to Fig. 1a). Next, they participated in a pre-test, where they viewed eight videos—six of the videos had errors embedded in them (Table 1)—while two were error-free. The participants were unaware of the type of videos presented. The participants were asked to assess the performances depicted in these videos using two assessment tools derived from the Objective Structured Assessment of Technical Skills (OSATS): Global Rating Scale (GRS) and Subcuticular Suture Checklist [10, 23, 24].

**Table 1:** Errors built into the six videos used in pre and post-tests

The needle holder is held with the thumb and index finger instead of the thumb and fourth finger	
The thumb is entirely inserted in the ring of the needle holder	
The needle is not held at two-thirds in the jaw of the needle holder	
Suturing is performed at an incorrect depth	
Excessive force is used with the forceps	
The needle is not inserted at a 90º angle	
Sutures are at inappropriate distances	
Extra unnecessary steps are performed	
The knot is not sufficiently tight	
Supination and pronation are not properly performed	
The thread is cut at an inappropriate length after tying the knot	
The thread is cut without seeing the knot	

#### Observational practice, collaboration and feedback

During this 3-day sub-phase, the participants were allowed to view a separate set of videos, as often as desired under one of the four experimental conditions. Control group: the participants viewed eight videos showing an expert performing a running subcuticular suture without errors. Self-learning: the participants viewed eight videos; six of these videos contained errors while two did not. In these two groups, they did not interact with any other participants. Peer-learning: the participants in this group also viewed eight videos, with six containing errors, and two error-free. However, they interacted with the other fourteen participants in this group for 3 days, and their task was to comment on the errors observed in the videos. The interactive format of GEN encouraged exchanges between participants in an anonymous fashion through the use of avatars (Fig. 1b). All participants were required to leave at least one comment for each video in order to proceed to the next phase of the study, and they could view each other’s comments and respond. Peer-learning with expert feedback: This group had the same conditions as the peer-learning group, however an expert provided comments and feedback to the group. The expert was a canadian-trained general surgeon and faculty member represented by a unique and easily identifiable-avatar.

#### Post-test

After a 3-day instructional period, all participants performed a post-test which consisted of the same eight videos and assessment tools presented in a different order.

#### Phase 2: Practical skills—performance test

Two weeks prior to the study, the simulators and instruments (Fig. 2) were sent to each participant. These simulators were used to conduct a test of performance, designed to measure how well the participants performed the skill on their first attempt after the instructional phase.

To accomplish this, after the post-test (i.e., test of procedural knowledge), the participants were instructed to record themselves opening a suturing kit and executing their first attempt at a running subcuticular suture. They uploaded their video on GEN and within a week, an expert surgeon assessed the participant’s performance in a blinded manner.

### Measurement tools

OSATS is a validated tool developed at the University of Toronto to assess surgical skills [19, 23, 24]. OSATS is composed of two parts. First is the GRS, by which global competencies are graded on a scale from 1 to 5 for a maximum possible total of 40 points (Table 2). The second is a checklist, which is a list of steps, and their order of execution, graded as dichotomous for a maximal score of 25 (Table 2).

**Table 2:** The OSATS’ Global Rating Scale (GRS) and the subcuticular suture checklist

Skill	1–2	3	4–5
Respect for tissue	Frequently used unnecessary force on tissues or caused damage by inappropriate instrument use	Careful handling of tissue, but occasional inadvertent damage	Consistently handled tissues appropriately with minimal damage
Time in motion	Many unnecessary moves	Efficient time and motion, but some unnecessary moves	Clear economy of movement and maximum efficiency
Instrument handling	Repeatedly makes tentative or awkward moves with instruments	Competent use of instruments, but occasionally awkward	Fluid movements
Suture skill	Awkward and unsure with poor knot tying, and inability to maintain tension	Competent suturing with good knot placement and appropriate tension	Excellent suture control with correct suture placement and tension
Flow of operation	Frequently stopped operating, seemed unsure of next move	Demonstrated some forward planning and reasonable progression of procedure	Obviously planned operation
Knowledge of procedure	Inefficient knowledge of procedure. Looked unsure and hesitant	Knew all important steps of procedure	Demonstrated familiarity of all steps of procedure
Final product	Final product of unacceptable quality	Final product of average quality	Final product of superior quality
Overall performance	Very poor	Competent	Very good

The Subcuticular Suture Checklist	
Check when correct, when properly done in the video. If N/A, leave unchecked	
**Appropriate use of suturing equipment**	
Needles and tissue always manipulated with equipment and not with hands	
Needle is properly held 2/3 from its tip	
Needle holder is appropriately held with thumb and fourth finger with extended index	
Forceps properly held with non-dominant hand	
Forceps are appropriately held like a pen	
**Execution of the running subcuticular suture**	
A deep dermal suture is performed on the extreme interior part of the wound	
At least three knots were performed with two loops around the needle holder the first one and 1 loop for the remaining knots	
The student alternate directions at 180 degrees angles parallel to the wound when tying the knot	
Thread after knot is cut at an appropriate length	
The deep dermal knot is not loose	
Needle is properly exited between the deep dermal knot and the apex of the wound	
Appropriate orientation of the needle upon insertion (at 90 degrees)	
For each subcuticular insertion the needle is properly inserted in the superficial layer of the dermis	
Each subcuticular insertion is appropriately spaced out and progresses through the wound	
Each bite is alternated on one side of the wound to the contralateral wound edge	
The surgical thread between each bite are parallel to each other	
Correct final knot technique performed with the needle holder. (Different techniques are permitted)	
The final knot is exited in line with the apex	
**Best practices**	
Supination and pronation of wrists	
Protection of the needle at the end of the intervention	
Surgeon can view the knot when it is cut and the scissors are held in a 45-degree angle	
**Final knot**	
Proper tightness of final knot	
Cut at an appropriate length	
**Overall aesthetics**	
Proper wound tension (no overlapping of tissues, no gaps)	
Thread is well beneath the surface of the skin	

### Data collection

#### Phase 1: The acquisition of procedural knowledge

The correct answers for the GRS and checklist were integrated into the GEN. Using the GRS and checklist, the participants were asked to identify correct and incorrect actions demonstrated within each video. If a mistake was present in the video but the participants checked the step as though it had been done properly, a point was deducted. Similarly, a point was deducted if a correctly executed step was not checked by the participant.

#### Phase 2: The initial hands-on performance

The expert surgeon used the same GRS and checklist to evaluate the suturing performance recorded by each participant uploaded to GEN.

### Statistical analyses

We followed the intention-to-treat (ITT) analysis, which includes each participant randomized according to randomized treatment assignment. This nullifies noncompliance, protocol deviations, withdrawal, and anything that happens after randomization [25]. Missing data for any of the tests resulted in the complete omission of the student’s data for statistical analysis. The Statistical Package for the Social Sciences [26] was employed for statistical analyses.

#### Phase 1: The acquisition of procedural knowledge

**Fig. 4:**
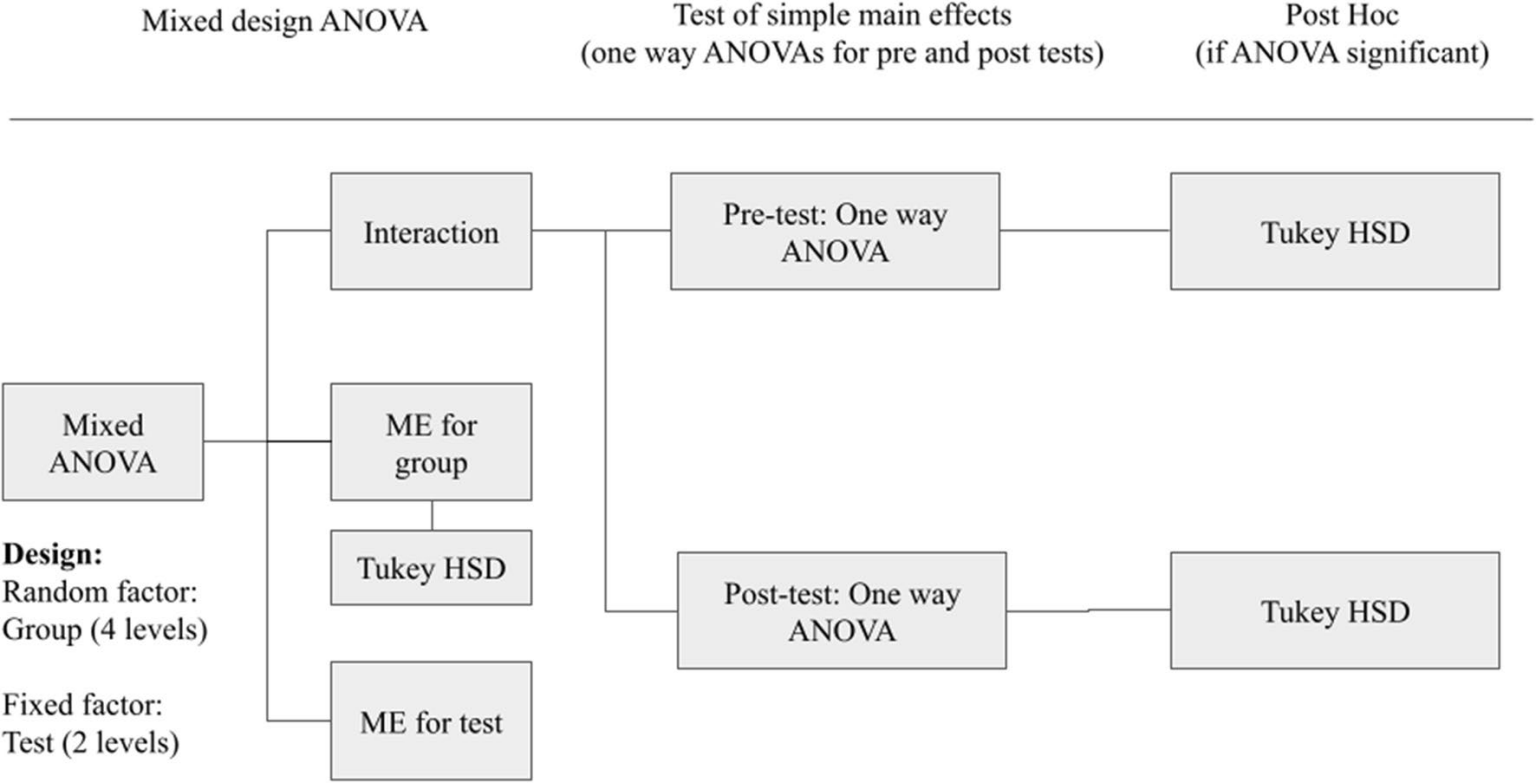
This figure shows the algorithm used for the statistical analysis in phase 1. Initially, a mixed-design ANOVA was used to test for main effects (ME) and interaction between group and test. If the interaction was found significant, we employed a set of tests of simple main effects in the form of a separate one-way ANOVAs for pre- and post-tests. For the significant one-way ANOVAs, we employed Tukey HSD test to test for group differences

Please refer to Fig. 4 for a graphic representation of the statistical analyses employed for phase 1. Initially, a separate mixed design analysis of variance (ANOVA) model with 4 groups (between subject factor) and 2 tests (within subject factor) was used to test the efficacy of the training method on procedural knowledge for GRS and checklists. Significant main effects at p < 0.05 were further analyzed using appropriate post hoc tests. However, if significant interaction between group and test was found, a set of simple main effects were used. We aimed to answer two questions: Were the groups similar or different in the pre-test? Were the groups similar or different in the post test? To achieve this, we used separate one-way ANOVAs for pre-test and post-test with a group as a single factor with four levels. All results that showed significance with *p* < 0.05 were further analyzed with the Tukey’s honestly significant difference (HSD) as a post-hoc analysis to compare the groups’ means and highlight differences.

#### Phase 2: The initial hands-on performance

A one-way, between groups ANOVA was used to test the efficacy of the training method on performance. Results were significant at *p* < 0.05 and were further analyzed with the Tukey’s honestly significant difference (HSD) as a post-hoc analysis to highlight differences.

## Results

### Phase 1: The acquisition of procedural knowledge

The data for one participant were excluded due to the lack of completion of the post-test (Self-learning group). The results for the GRS and checklist were analyzed separately and are presented in Table 3.

**Table 3:** This table shows the averages and standard deviations in the GRS and checklists between the four groups as a function of testing

Group	Global rating scores				Checklist			
	Pre-test		Post-test		Pre-test		Post-test	
	Mean	SD	Mean	SD	Mean	SD	Mean	SD
Control	18.3	7.88	26.4	9.67	17.2	2.95	21.9	2.63
Self-learning	14.5	6.30	18.5	5.00	17.6	2.94	20.1	1.43
Peer-learning	16.1	4.80	18.5	4.30	19.0	1.35	20.8	1.12
Peer-learning with expert feedback	14.3	4.80	17.2	5.00	18.5	2.55	21.0	1.20

#### Global rating scales

In summary, the results showed that the control group identified more errors within the videos on the post-test as compared to the other three groups. Specifically, the Tukey HSD tests revealed that the Control group showed higher scores on the post-test than Self-learning group (p = 0.008), Peer-learning (p = 0.007), and the Peer-learning with expert feedback (p = 0.001) groups.

Firstly, the mixed design ANOVA revealed a significant interaction between group and test (F = 2.87, p = 0.044). Subsequently, simple main effects were used to determine the difference between the four groups at each level of the test variable (i.e., pre-test and post-test). These analyses showed that there were no significant differences between the four groups at pre-test (F(3,55) = 1.39, p = 0.255). In contrast, there was a significant difference between the four groups during the post-test (F(3,55) = 6.649, p < 0.001). Tukey HSD tests revealed that the Control group showed higher scores on the post-test than Self-learning group (p = 0.008), Peer-learning (p = 0.007), and the Peer-learning with expert feedback (p = 0.001) groups. None of the other three groups showed significant difference during the post-test at p = 0.05.

#### Checklist

The results showed that the control group identified more errors within the videos on the post-test as compared to the other three groups. Specifically, the Tukey HSD tests revealed that the Control group showed higher scores on the post-test than Self-learning group (p = 0.033), but not from Peer-learning (p = 0.323), and the Peer-learning with expert feedback (p = 0.458) groups.

Firstly, the mixed design ANOVA revealed a significant interaction between group and test (F(3,55) = 7.189, p < 0.001). Simple main effects showed that there were no significant differences between the four groups during the pre-test (F(3,55) = 1.677, p = 0.188). In contrast, there was a significant difference between the four groups during the post-test (F(3,55) = 2.696, p = 049). Tuckey HSD tests revealed that the Control group showed higher scores on the post-test than Self-learning group (p = 0.033), but not from Peer-learning (p = 0.323), and the Peer-learning with expert feedback (p = 0.458) groups. None of the other three groups showed significant difference between each other on this measure during the post-test at p = 0.05.

#### Phase 2: Practical skills—performance test

The data for two participants in the *Self-learning group*, two participants in the *Peer-learning group*, and one participant in the *Peer-learning with expert group* were excluded due to the lack of completion of this test. The results for the GRS and skill specific checklist were analyzed separately, and are presented in Table 4.

**Table 4:** This table shows the averages and standard deviations in the GRS and checklists between the four groups

Group	Global rating scores		Checklist	
	Mean	SD	Mean	SD
Control	18.7	6.75	12.1	4.27
Self-learning	18.0	4.00	12.7	1.97
Peer-learning	26.5	7.24	16.4	4.81
Peer-learning with expert feedback	25.7	6.99	18.1	3.51

#### Global rating scales

The one-way between group ANOVA revealed a significant main effect (F(3,54) = 6.70, p = 0007). Pairwise comparisons showed that the Control and Self-learning groups did not differ significantly from each other (t = −0.27, p = 0.993), and the Peer-learning and Peer-learning with expert feedback group did not differ from each other (t = −0.30, p = 0.990). On the contrary, when contrasted with the Control group both the Peer-learning group (t = 3.21, p = 0.012) and the Peer-learning with expert group (t = 2.96, p = 0.023) showed higher scores. Similarly, when contrasted with the Self-learning group both the Peer-learning group (t = 3.37, p = 0.008) and the Peer-learning with expert group (t = 3.12, p = 0.015) showed higher scores.

#### Checklist

The one-way between group ANOVA revealed a significant main effect (F(3,54) = 8.13, p = 0002). Pairwise comparisons showed that the Control and Self-learning groups did not differ significantly from each other (t = 0.39, p = 0.980), and the Peer-learning and Peer-learning with experts groups did not differ from each other (t = 1.20, p = 0.630). On the contrary, when contrasted with the Control group both the Peer-learning group (t = 2.95, p = 0.024) and the Peer-learning with expert group (t = 4.25, p = 0.001) showed higher scores. Similarly, when contrasted with the Self-learning group the Peer-learning with expert group (t = 3.72, p = 0.003) showed higher scores. When contrasted with the Self-learning group, the Peer-learning group also only showed trends towards achieving higher scores (t = 2.47, p = 0.077).

## Discussion

The concept of a decentralized model of simulation (DeC-SIM) is not new [2], however, the recent COVID-19 pandemic catalyzed vast research and development efforts in this area [3, 4]. Based on Ericsson’s theory of deliberate practice [5], to ensure the effectiveness and subsequent consideration of DeC-SIM as a possible adjunct to more traditional training approaches (i.e., C-SIM), the initial work should focus on creating a set of best practices for designing basic simulation elements such as instructions, scheduling and monitoring remote practice, maintaining learners’ motivation, and providing accurate feedback [1, 4, 5]. In this study, we have focused on how to structure instructions in a DeC-SIM model to (a) most optimally develop procedural knowledge prior to physical practice, and (b) improve learners’ performance during the initial hands-on practice.

The results of phase 1 showed that all learners improved their procedural knowledge of the suturing technique, becoming more familiar with the suturing task and the assessment tools. On the pre-test, all learners scored similarly, while on the post-test, the learners in the control group had a higher result, although this may not necessarily be interpreted as representative of a superior performance. The learners in the control group observed and assessed a set of eight error-free videos, while those in the other three groups observed and assessed videos with built-in errors; making the videos of the control group easier to assess. Overall, the shift in the ability to discern error-free and erroneous videos from pre-test to post-test implies that the observational practice was effective.

Phase 2 of this study aimed to address whether the conditions of observational practice led to different psychomotor performances on the very first attempt at hands-on practice. This was based on Miller’s model [22] which proposes that the degree of procedural knowledge and the degree of competence, or what we refer to as the first attempt at psychomotor performance, may not always match. Although typically research shows a gap in transfer of procedural knowledge to competent performance [27], we wanted to test the opposite hypothesis—that although the various conditions of observational practice and collaboration lead to similar procedural knowledge, they may have a differential impact on initial motor performance.

The results of phase 2 suggest that collaborative, peer learning conditions lead to procedural knowledge that translates to an improved initial motor performance compared to similar practice in isolation. Furthermore, observing error-free videos vs those with errors during the observational practice, did not impact the psychomotor performance. Most importantly, however, the presence of an expert in the collaborative, peer-learning group did not affect the initial motor performance.

Collectively, our results are in support of the idea of ‘preparation for future learning' [28]. More specifically, these results indicate that DeC-SIM is a feasible addition to the current laboratory-based simulation learning model. For this approach to be optimal, virtual learning management systems, such as GEN, must support collaborative, peer-learning approaches [2, 13, 14, 29]. One key finding stemming from the current study, is that the addition of an expert in a collaborative, peer-learning group does not impact the development of procedural knowledge or subsequent motor performance. The fact that the presence of an expert did not lead to better learning outcomes may have a practical implication for future adoption of DeC-SIM by relevant stakeholders and policymakers.

Although promising, the study has several limitations that should be acknowledged. First, the experimental design used was not orthogonal. In our context, orthogonality refers to the property of the experimental design that ensures that all conditions of practice may be studied independently. Instead, in this exploratory, pilot randomized control study, a planned comparison design approach was used. Because of the exploratory nature of this work, and the aim of testing a complex intervention, our focus was on a few comparisons of interest rather than every possible comparison. Future work will emphasize the need for more orthogonal designs. Secondly, the participants’ satisfaction with the learning environment was not assessed. According to Kirkpatrick’s model [30], the participant’s experience should be evaluated and may provide approximate levels of acceptability of the new training approach by the end point users. In addition, based on the principles of Utilization-Focused Evaluation (U-FE) [31] such assessment of satisfaction may also provide early evidence of the areas of improvement of the intervention. Also, for the performance test, future studies should consider additional raters in order to have a reliable and stable assessment [32]. Finally, we only investigate the effectiveness of the DeC-SIM when applied to the acquisition of fundamental surgical skills by naive or novice learners. In accordance with contemporary progressive learning frameworks [33], future work should extend our current findings to more complex skills and more advanced learners.

In summary, the current results fit well with prior evidence on this topic, and suggest that junior surgical learners are effective at using video-based instructions for preparation [12], and that creating opportunities for peer-to-peer collaboration [13, 14], with and without an expert [2, 15], can further facilitate preparation and instructions for subsequent hands-on practice. However, to the best of our knowledge, this is the first study to include a set of instructional elements to form a complex simulation intervention that would support DeC-SIM model in the future.
